# An Ice Vest, but Not Single-Hand Cooling, Is Effective at Reducing Thermo-Physiological Strain During Exercise Recovery in the Heat

**DOI:** 10.3389/fspor.2021.660910

**Published:** 2021-04-29

**Authors:** Afton D. Seeley, Ross A. Sherman

**Affiliations:** ^1^Department of Human Performance and Health Education, Western Michigan University, Kalamazoo, MI, United States; ^2^Thermal and Mountain Medicine Division, US Army Research Institute of Environmental Medicine, Natick, MA, United States; ^3^Oak Ridge Institute of Science and Education, Belcamp, MD, United States; ^4^Department of Movement Science, Grand Valley State University, Allendale, MI, United States

**Keywords:** exercise in heat, thermoregulation, post-exercise recovery, skin temperature, heat storage, core temperature, sport

## Abstract

Sports limit the length of breaks between halves or periods, placing substantial time constraints on cooling effectiveness. This study investigated the effect of active cooling during both time-limited and prolonged post-exercise recovery in the heat. Ten recreationally-active adults (VO_2peak_ 43.6 ± 7.5 ml·kg^−1^·min^−1^) were exposed to thermally-challenging conditions (36°C air temperature, 45% RH) while passively seated for 30 min, cycling for 60 min at 51% VO_2peak_, and during a seated recovery for 60 min that was broken into two epochs: first 15 min (REC_0−15_) and total 60 min (REC_0−60_). Three different cooling techniques were implemented during independent recovery trials: (a) negative-pressure single hand-cooling (~17°C); (b) ice vest; and (c) non-cooling control. Change in rectal temperature (*T*_re_), mean skin temperature (${\bar{T}}_{\text{sk}}$), heart rate (HR), and thermal sensation (TS), as well as mean body temperature (${\bar{T}}_{\text{b}}$), and heat storage (*S*) were calculated for exercise, REC_0−15_ and REC_0−60_. During REC_0−15_, HR was lowered more with the ice vest (−9 [−15 to −3] bts·min^−1^, *p* = 0.002) and single hand-cooling (−7 [−13 to −1] bts·min^−1^, *p* = 0.021) compared to a non-cooling control. The ice vest caused a greater change in ${\bar{T}}_{\text{sk}}$ compared to no cooling (−1.07 [−2.00 to −0.13]°C, *p* = 0.021) and single-hand cooling (−1.07 [−2.01 to −0.14]°C, *p* = 0.020), as well as a greater change in *S* compared to no cooling (−84 [−132 to −37] W, *p* < 0.0001) and single-hand cooling (−74 [−125 to −24] W, *p* = 0.002). Across REC_0−60_, changes in ${\bar{T}}_{\text{b}}$ (−0.38 [−0.69 to −0.07]°C, *p* = 0.012) and ${\bar{T}}_{\text{sk}}$ (−1.62 [−2.56 to −0.68]°C, *p* < 0.0001) were greater with ice vest compared to no cooling. Furthermore, changes in in ${\bar{T}}_{\text{b}}$ (−0.39 [−0.70 to −0.08]°C, *p* = 0.010) and ${\bar{T}}_{\text{sk}}$ (−1.68 [−2.61 to −0.74]°C, *p* < 0.0001) were greater with the ice vest compared to single-hand cooling. Using an ice vest during time-limited and prolonged recovery in the heat aided in a more effective reduction in thermo-physiological strain compared to both passive cooling as well as a single-hand cooling device.

## Introduction

Both professional and recreational sporting events frequently take place in thermally-stressful environments, including the familiar summer Olympic Games (Barwood et al., 2009). Unabated heat gain during exercise performed in hot environments, the product of both elevated muscular work and a reduced skin temperature to ambient temperature (*T*_sk_-*T*_a_), and therefore core temperature to skin temperature (*T*_c_-*T*_sk_), thermal exchange gradient, is capable of eliciting hyperthermic core body temperatures (*T*_c_) >39°C (Wendt et al., 2007). Heated exercise-induced core and skin temperatures elevations contribute to the deterioration of aerobic exercise capacity, although the exact cardiovascular mechanisms by which this decrement occurs appears to be a function of both exercise intensity and duration (Nybo et al., 2011). At maximal exercise intensities, augmentation of skin blood flow to facilitate heat loss impairs cardiac filling, reducing central venous pressure, and maximal cardiac output, and therefore competitively impairs arterial oxygen delivery to exercising skeletal muscle. At prolonged submaximal intensities when muscle blood flow and oxygen consumption are often not significantly changed relative to a more temperate environment, high skin temperatures likely influence the perception of fatigue via alterations in afferent feedback (Nybo et al., 2011). Continually increasing core and skin temperatures, especially during prolonged exercise, additionally pose an enhanced likelihood of heat illness. Heat stroke, the most serious heat related syndrome, is designated by a severely elevated core temperature and failure of an individual's sweating mechanisms (Coris et al., 2004).

Athletes that participate in outdoor sporting events of a prolonged heated nature, such as ultramarathons, and those with a limited 10–15 min half-time, such as soccer and rugby, may be especially susceptible to the deleterious influence of hyperthermia. Strategic pre-exercise behaviors including heat acclimation (Lorenzo et al., 2010) and intra-exercise hydration strategies (Montain and Coyle, 1992; Travers et al., 2020) have proven helpful to reduce the physiological stress and performance decrement induced by hyperthermia. Furthermore, athletes engaging in prolonged or limited breaking sport have the potential to both offset aerobic performance decrement as well as the risk of heat illness by implementing efficient cooling strategies available during sporting breaks. While application of cooling devices in a continually heated environment may influence overall cooling effectiveness, sport, and occupational requirements often impede the removal of a hyperthermic individual from a heated environment. Furthermore, the implementation of techniques such as whole-body cold water immersion, deemed to be most efficient for thermoregulatory recovery (Casa et al., 2015; Zhang et al., 2015), is practically difficult in prophylactic cooling scenarios. Additionally, neck cooling, although effective in reducing the perception of heat strain (Sunderland et al., 2015), does not appear to meaningfully assist in thermo-physiological rebound from hyperthermia (Tyler and Sunderland, 2011; Sunderland et al., 2015). Therefore, using more practical and physiology-influencing cooling techniques during heated endurance exercise recovery, if deemed time-efficient in their cooling, may help to offset decrement of discontinuous aerobic exercise performance and the accumulated risk of severe heat illness.

Numerous commercially available small and portable cooling devices have been proposed to reduce cardiovascular strain, skin temperature, and core temperature following exercise-induced hyperthermia.

A phase-changing “ice” vest provides a widened *T*_c_-*T*_sk_ gradient, by reducing skin temperature, which favors heat loss from the blood perfusing the skin. The cooled blood then circulates back to the core, effectively contributing to the maintenance or development of a negative heat balance (House et al., 2013). The donning of an ice vest in occupational situations beneath clothing has effectively demonstrated reductions in heat strain (House, 1996; Cadarette et al., 2002; House et al., 2003; Amorim et al., 2010). The use of ice vests by hyperthermic athletes post-exercise, though, has proven less successful in reducing core temperature, with studies citing a loss in evaporative cooling with the torso encompassing vests (House et al., 2013).

Additional post-exercise cooling efforts have monopolized on hand cooling technologies some of which utilize a rigid chamber with a flexible airtight vacuum-seal about the wrist. Hand cooling is thought to optimize the loss of body heat through the arteriovenous anastomoses (AVAs) present in the palm, effectively dissipating heat at elevated core and skin temperatures (Bergersen, 1993). Battery powered cooling devices create a thermal gradient with the palm while the negative pressure in the vacuum chamber draws a large volume of blood into the AVAs to speed heat exchange and prevent cold-reactive vasoconstriction (Zhang et al., 2009). Research has focused largely on the use of this relatively light and portable hand cooling device during exercise (Grahn et al., 2005, 2012; Hsu et al., 2005), with only a small and inconclusive body of support for single-hand use in recovery (Zhang et al., 2009; Kuennen et al., 2010). One of the most recent investigations, using a commercially available hand cooling device (Adams et al., 2016) following heated treadmill exercise, suggests that dual hand cooling may reduce *T*_re_ more than passive cooling alone, bolstering similar conclusions drawn from dual hand immersion in a simpler cold water bath (Barwood et al., 2009). Yet still, a variety of literature suggests a lack of advantageous core temperature reduction with administration of a single hand cooling device during exercise recovery (Balldin et al., 2007; Walker et al., 2009; Amorim et al., 2010).

Existing literature suggests augmented cooling by single-hand cooling when heavy heat retardant clothing is worn/retained following heated exercise and into heated recovery (Kuennen et al., 2010) and/or when hand cooling is imposed simultaneously with removal from the heated environment (Zhang et al., 2009). Furthermore, dual hand cooling, although much more logistically burdensome to dexterity, appears efficacious to efficiently reduce core temperature following heated exercise, likely due to the heightened cooling exposed skin surface area (Barwood et al., 2009; Adams et al., 2016). Still, little literature exists regarding the influence of a negative pressure hand cooling device on a sport-mimicking recovery environment where minimal clothing (shorts and t-shirt) is worn, individuals are not removed from the heated environment following exercise, and only single-hand cooling is utilized to retain dexterity.

The primary aim of this study is to investigate the effectiveness of active vs. passive cooling during time-limited and prolonged recovery in sport-mimicking conditions (minimal clothing, continued exposure to heat, dexterity maintenance) following submaximal exercise in the heat. A secondary aim of this study is to examine thermo-physiological and perceptual differences during recovery in the heat between negative pressure single-hand cooling and an ice vest. We hypothesized that active post-exercise cooling would significantly improve thermo-physiological function and perceived thermal sensation (TS) in both time-limited and prolonged recovery. Additionally, it was proposed that an ice vest would be a more effective cooling strategy than single-hand cooling.

## Materials and Methods

### Participants and Procedural Controls

Ten recreationally active participants (six males, four females, age 25 ± 3 years, body mass 75.5 ± 12.5 kg, height 173 ± 9 cm, VO_2peak_ 43.6 ± 7.5 ml·kg^−1^·min^−1^) participated in the study. All participants met the following inclusionary criteria: non-smoker; healthy, free of disease, and free of medication use which may affect the cardiovascular or metabolic responses during exercise; free of any orthopedic injuries or conditions that would make exercise difficult; classified as “Low Risk” by the American College of Sport Medicine (Pescatello et al., 2014); and not obese (body mass index <30 kg·m^−2^). Participants gave written, informed consent prior to participation in the study, which had been approved by the Human Subjects Institutional Review Board (Project Approval Number: 13-12-10) at Western Michigan University.

Participants were asked to refrain from ingesting any caffeine and engaging in exercise the day of the visits to the laboratory. Eumenorrhoeic female participants were asked to self-report the date of last menses to restrict collection of thermoregulatory data only within the early follicular phase (cycle days 3–6). Those that reported using a biphasic oral contraceptive (*n* = 1) were tested within the first 3–6 days of active pills following the withdrawal week, in an attempt to minimize the day-to-day variability in thermoregulatory variables, especially rectal temperature (Lei et al., 2019). Notably, pre-exercise rectal temperature did not significantly differ between conditions (control: 37.13 ± 0.30, ice vest: 37.23 ± 0.28, single-hand cooling: 37.17 ± 0.30, *p* = 0.734). Each participant was asked to wear a t-shirt, shorts, socks, and athletic shoes each time they visited the laboratory. All trials for a given participant were conducted during the morning (± 1 h) to avoid diurnal variation in core temperature (Morris et al., 2009). The research was conducted outside of the summer months (September–April) in order to minimize any seasonal heat acclimation. Furthermore, all heated trials were separated by at least 1 week to minimize the likelihood of induced heat acclimation.

### Research Design

The study was conducted utilizing a randomized counterbalanced cross-over design with three recovery conditions: (1) negative pressure single-hand cooling, (2) ice vest, and (3) a non-cooling control. Participants visited the laboratory on four separate occasions. The first visit consisted of a graded exercise test and the following three visits consisted of exercise bouts in the heat followed by one of the three recovery cooling conditions.

### Graded Exercise Test

Upon arrival to the laboratory, height and body mass were measured using standardized techniques and a wall-mounted stadiometer and digital scale, respectively. Each participant completed a graded exercise test on an electromagnetically-braked cycle ergometer (Corival, Lode B.V., Groningen, Netherlands) to determine peak oxygen consumption (VO_2peak_) as a measure of cardiorespiratory fitness. The VO_2peak_ value obtained allowed determination of the appropriate exercise intensity (~50% VO_2peak_) for all experimental trials. Each participant was fitted for seat height on the cycle ergometer, with the participant's knee at 10–15° of flexion at the pedal's lowest point. Additionally, each participant was fitted with a nose clip and a mouthpiece for the collection of 15-s averaged expired respiratory gases using a metabolic cart (TrueOne 2400, ParvoMedics, Sandy, UT), and a heart rate (HR) monitor (Polar USA, Lake Success, Long Island, NY). The assessment consisted of a graded protocol that began with two- min of cycling at 40 W for female and 60 W for male participants. The cycling intensity was increased every minute thereafter by 20 W until volitional fatigue. Volitional fatigue was determined as the point during exercise when each participant felt like they could exercise no longer or could no longer maintain a pedaling frequency of at least 50 rpm. Each participant was asked to assess their ratings of perceived exertion (RPE) using a standard 6–20 scale (Borg and Linderholm, 1967) during the last 30 s of each stage. Once the exercise test protocol was terminated, each participant was provided water *ad libitum* and continued to cycle at a low intensity for 5–10 min whilst being monitored for normal, post-exercise cardiovascular recovery.

### Experimental Trials

Upon arrival at the laboratory, participants' nude body mass was measured. Thirty minutes prior to entering the heated (36°C, 45% RH) environmental chamber (Thermotron, Holland, MI) a bolus of plain water equivalent to 5 ml·kg^−1^ body mass was administered in an attempt to standardize pre-exercise hydration status. A flexible probe (Physitemp Instruments Inc., Clifton, NJ) was inserted 13 cm past the anal sphincter for the measurement of rectal temperature (*T*_re_). Thermocouples (Physitemp Instruments Inc., Clifton, NJ) were also attached to the surface of the skin at four sites on the right side of the body (chest, triceps, quadriceps, calf) using waterproof tape (Hy-tape, Hytape International Inc., Patterson, NY) for the measurement of skin temperature (*T*_sk_). The four sites contributed to the calculation of mean skin temperature (${\bar{T}}_{\text{sk}}$; Ramanathan, 1964):

$$\begin{array}{l} {\bar{T}}_{\text{sk}}=0.3\left(T_{\text{chest}}+T_{\text{arm}}\right)+0.2\left(T_{\text{thigh}}+\;T_{\text{calf}}\right) \end{array}$$

Both *T*_re_ and ${\bar{T}}_{\text{sk}}$ were then used to calculate mean body temperature (${\bar{T}}_{\text{b}}$; Colin and Houdas, 1965):

$$\begin{array}{l} {\bar{T}}_{\text{b}}=\left(0.8\cdot {\text{T}}_{\text{re}}\right)+\left(0.2\cdot {\bar{T}}_{\text{sk}}\right) \end{array}$$

Stored heat (*S*) was calculated for the exercise bout and recovery using the following equation:

$$\begin{array}{l} S=\Delta {\bar{T}}_{\text{b}}\cdot 3.48\cdot mass/t \end{array}$$

where: $\Delta {\bar{T}}_{\text{b}}$ = change in mean body temperature, the average specific heat of body tissues was assumed as 3.48 kJ·kg^−1^·°C^−1^, mass = pre-test mass of the participant (kg), *t* = time (s).

Rectal and skin thermocouples were connected to a data acquisition system (Thermes USB, Physitemp Instruments Inc., Clifton, NJ) that was interfaced to a PC computer. Lastly, a HR monitor was fitted.

Each trial consisted of the following sequence performed entirely within the environmental chamber at 36°C, 45% RH:

- 30 min of seated rest
- 60 min of cycling at ~50% VO_2peak_ or until *T*_re_ ≥39.5°C
- 60 min of seated rest with one of the cooling conditions.

Time to complete this trial sequence within the environmental chamber across all conditions was 154.12 ± 1.26 min. Each participant's target exercise VO_2_ and starting power output was determined utilizing the established power output to VO_2_ relationship determined using the VO_2peak_ assessment. During each participant's first trial, expired respiratory gases were collected during the first 10 min of exercise. If the 15-s averaged VO_2_ from 5:15 to 7:00 after the start of exercise was not 50 ± 5% VO_2peak_, the intensity was adjusted in 5 W increments until target VO_2_ was reached. Exercise intensity was monitored for an additional 2 min upon readjustment to ensure VO_2_ stability. The progression of exercise intensity for each subsequent trial followed an identical scheme: (1) 2-min warm-up at half of the 50% VO_2peak_ power output, (2) at 2 min, the intensity was increased to the 50% VO_2peak_ eliciting power output, and if needed (3) power output was adjusted from 7 to 10 min of exercise.

Both *T*_re_ and *T*_sk_ were continuously monitored throughout all phases of each trial. *T*_re_ and *T*_sk_ were recorded during the last 5 min of passive rest, and every 5 min during exercise and recovery. Participants were also asked to assess their TS using a standard 0–8 scale (Gagge et al., 1967) during the last 5 min of passive rest, and every 5 min during exercise and recovery. Participants were prevented from ingesting any fluids throughout the full duration of each trial (start of passive rest through to the end of recovery). Nude body mass was again measured immediately after the end of recovery in each trial to allow for the determination of fluid loss via change in nude body mass.

### Recovery Phase

The 60 min post-exercise seated rest portion of the study (REC_0−60_), where each of the three cooling interventions was applied while within 36°C, 45% RH conditions based on the randomized order, was split into two epochs; the first time-limited, 15 min recovery period (REC_0−15_) and the prolonged 60 min recovery period (REC_0−60_) to allow for short- and long-term responses to be monitored and assessed. The control condition consisted of passive seated recovery following exercise cessation and had participants sit quietly with minimal movement in a backed chair with their feet planted on the floor.

### Hand Cooling

The hand cooling device (CoreControl, AVAcore Technologies, Ann Arbor, MI) was administered with participants seated in a backed chair, and fixed to the dominant hand and forearm. Participants were instructed to place their hand over the small soft disc at the bottom of the device to ensure standardized exposure. Using a water-dwelling thermometer, the temperature of the continuously perfusing water was maintained at ~17°C with the addition of more ice to the slurry mix when necessary. The device also used a low pressure (~15 mmHg) vacuum around the forearm to facilitate blood transport through the AVAs and prevent acute vasoconstriction. This low pressure vacuum was maintained for the duration of the exposure.

### Ice Vest

The ice vest (Kool Max Poncho Vest, Polar Products, Stow, Ohio) was adjustable to fit all torso sizes, and was equipped with frozen ice packs (Kool Max Ice Packs, Polar Products, Stow, Ohio) fixed in 10 individual pockets dispersed equally on the front and the back of the torso. Upon initial application, the temperature of the packs was 0°C with no attempt to control heat gain over the duration of application. Participants were seated in a backed chair with their feet planted on the floor.

### Statistical Analysis

A one-way analysis of variance (ANOVA) was used to assess exercise differences in VO_2_, pre-post trial nude body mass, as well as change in ${\bar{T}}_{\text{b}}$, *S, T*_re,_ ${\bar{T}}_{\text{sk}}$, HR, and TS across the three experimental exercise trials. Normality of dependent variables was assessed using a Shapiro-Wilk test and sphericity for each main and interaction effect using Mauchly's sphericity test. Delta data for ${\bar{T}}_{\text{b}}$, *S, T*_re,_ ${\bar{T}}_{\text{sk}}$, HR, and TS from recovery baseline values were analyzed using linear mixed modeling with restricted maximum likelihood and Satterthwaite small-sample correction of degrees of freedom. The model had fixed factors of condition (control, ice vest, single-hand cooling), time (REC_0−15_, REC_0−60_), and condition*time with a covariate (value at start of recovery) equal across conditions. The model included a random intercept by participant to account for the hierarchical data structure (repeated measures within participants). A secondary linear mixed model with baseline recovery covariate was conducted to further investigate REC_0−15_ components (REC_0−5_, REC_5−10_, REC_10−15_) across conditions. For all statistical procedures, when appropriate, *post-hoc* pairwise comparisons were conducted using a Sidak correction to reduce Type I error. Statistical significance was set at *p* < 0.05 for all analyses, and data was analyzed using IBM SPSS version 26.0 (IBM Corporation, Chicago, IL). All absolute data are presented as mean ± standard deviation and all delta data are presented as mean difference with 95% confidence intervals where appropriate.

## Results

### Exercise in the Heat

The VO_2_ averaged across all exercise bouts was 1.65 ± 0.42 L·min^−1^ (51 ± 2% VO_2peak_), which was elicited by 92 ± 35 W. No differences in VO_2_ existed between experimental trials (*p* = 0.483). Thermo-physiological responses were not different across the three exercise bouts in the heat, with similar heat storage (control: 89 ± 35 W, ice vest: 91 ± 32 W, single hand-cooling: 82 ± 26 W; *p* = 0.297) and end-exercise *T*_re_ (control: 38.28 ± 0.31°C, ice vest: 38.37 ± 0.24°C, single hand-cooling: 38.22 ± 0.40°C; *p* = 0.250) across the three trials.

### Post-exercise Recovery in the Heat

#### Thermoregulation

**Figure 1:**
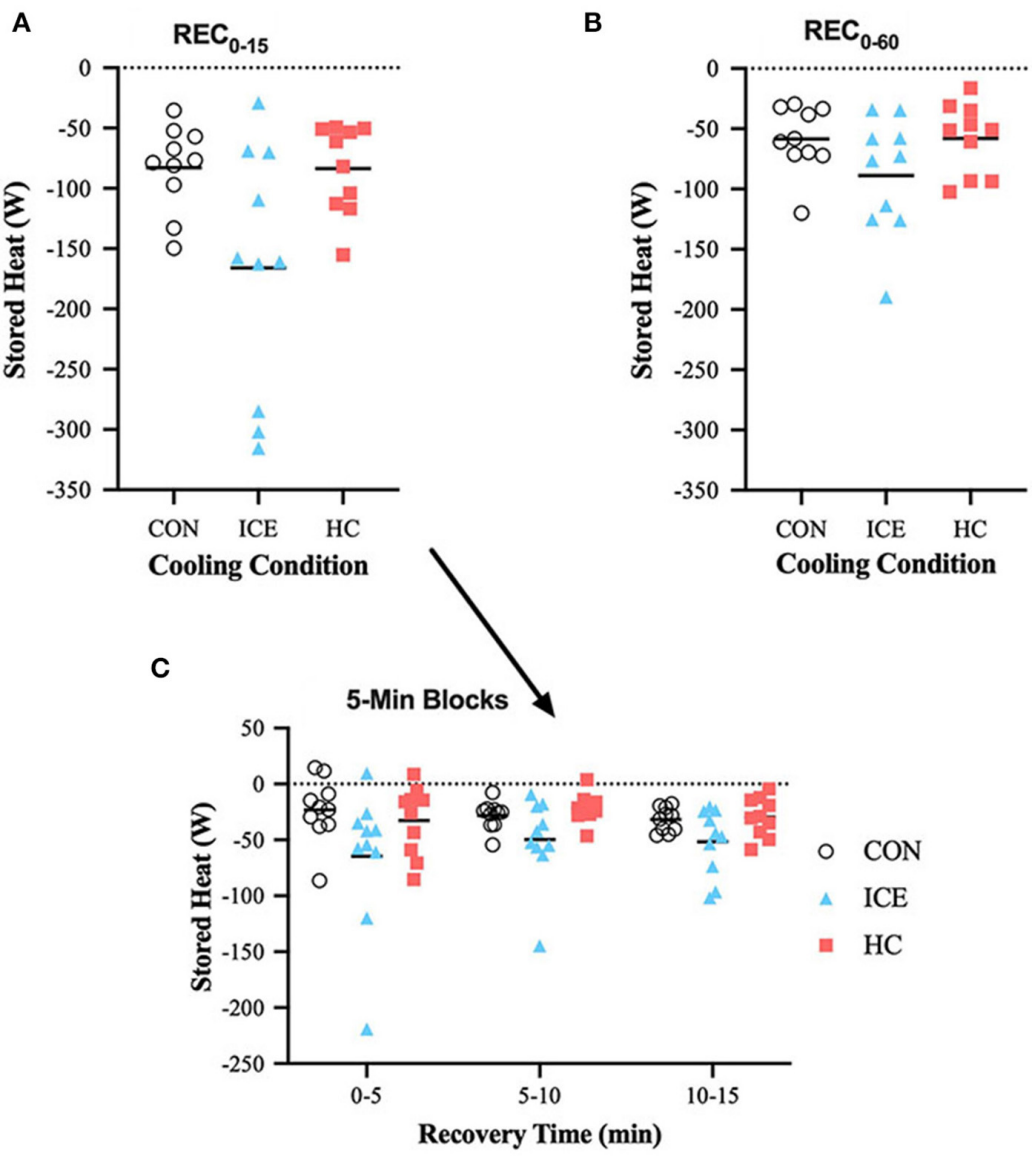
Mean (bar) and individual delta stored heat (*S*) during REC_0−15_ **(A)**, REC_0−60_ **(B)**, and 5-min blocks during REC_0−15_ **(C)** when using ice vest (ICE), single-hand cooling (HC), and non-cooled control (CON) during heated exercise recovery.

Stored heat (S) reduction during REC_0−15_ was different between cooling conditions, with the application of an ice vest eliciting a significantly greater reduction of stored heat across time-limited recovery (REC_0−15_) compared to a non-cooling control (−84 [−132 to −37] W, *p* < 0.0001) and single-hand cooling (−74 [−125 to −24] W, *p* = 0.002). A deeper look into the components of time-limited recovery indicates that heat loss was increased across the first 5 min of ice vest application when compared to both control (−44 [−73 to −15] W, *p* = 0.001) and single-hand cooling (−31 [−61 to −0.3] W, *p* = 0.047). Specifically for the ice vest recovery condition, the rate of heat loss was greater during the time-limited recovery than during prolonged recovery (−69 [−107 to −31] W, *p* = 0.001). Mean delta and individual data for S can be found in Table 1 and Figure 1, respectively.

**Table 1:** Delta mean [95% confidence interval] rectal temperature (*T*_re_), skin temperature (${\bar{T}}_{\text{sk}}$), body temperature (${\bar{T}}_{\text{b}}$), stored heat (*S*), heart rate (HR), and thermal sensation (TS) during prolonged recovery (REC_0−60_), and time-limited recovery (REC_0−15_) with 5 min block component analysis (REC_0−5_, REC_5−10_, REC_10−15_) when using a non-cooled control, ice vest, and single hand-cooling during heated exercise recovery.

	**Recovery Time (min)**	**Control**	**Ice Vest**	**Single-Hand Cooling**	**Condition**	**Time**	**Interaction**
*T*_re_ (°C)	0–5	−0.05 [−0.10 to 0.003]	−0.03 [−0.08 to 0.03]	−0.07 [−0.12 to −0.02]	*p = 0.619*	***p = 0.037***	*p = 0.252*
	5–10	−0.07 [−0.13 to −0.02]	−0.11 [−0.16 to −0.06]^a^	−0.06 [−0.11 to −0.01]			
	10–15	−0.09 [−0.14 to −0.04]	−0.13 [−0.18 to −0.08]^a^	−0.08 [−0.13 to −0.03]			
	0–15	−0.21 [−0.39 to −0.03]^b^	−0.26 [−0.44 to −0.09]^b^	−0.21 [−0.39 to −0.04]^b^	*p = 0.673*	***p < 0.0001***	*p = 0.990*
	0–60	−0.66 [−0.84 to −0.49]	−0.73 [−0.91 to −0.56]	−0.66 [−0.84 to −0.49]			
${\bar{T}}_{\text{sk}}$ (°C)	0–5	−0.22 [−0.44 to −0.001]	−0.96 [−1.18 to −0.74]^*^^†^	−0.27 [−0.49 to −0.05]	***p < 0.0001***	***p = 0.010***	***p = 0.039***
	5–10	−0.20 [−0.42 to 0.02]	−0.37 [−0.59 to −0.15]^a^	−0.14 [−0.36 to 0.08]			
	10–15	−0.19 [−0.41 to 0.03]	−0.35 [−0.57 to −0.13]^a^	−0.19 [−0.41 to 0.03]			
	0–15	−0.61 [−1.17 to −0.04]	−1.67 [−2.24 to −1.10]^*^^†^^*b*^	−0.60 [−1.17 to −0.03]	***p < 0.0001***	***p < 0.0001***	*p = 0.463*
	0–60	−1.34 [−1.91 to −0.77]	−2.96 [−3.53 to −2.39]^*^^†^	−1.28 [−1.85 to −0.71]			
${\bar{T}}_{\text{b}}$ (°C)	0–5	−0.08 [−0.14 to −0.02]	−0.21 [−0.27 to −0.15]^*^	−0.11 [−0.17 to −0.05]	***p = 0.001***	*p = 0.611*	*p = 0.755*
	5–10	−0.10 [−0.16 to −0.04]	−0.16 [−0.22 to −0.10]	−0.08 [−0.14 to −0.02]			
	10–15	−0.11 [−0.17 to −0.05]	−0.17 [−0.23 to −0.11]	−0.10 [−0.16 to −0.04]			
	0–15	−0.29 [−0.50 to −0.08]^b^	−0.54 [−0.75 to −0.34]^b^	−0.29 [−0.50 to −0.08]^b^	***p = 0.001***	***p < 0.0001***	*p = 0.697*
	0–60	−0.80 [−1.01 to −0.59]	1.18 [−1.39 to −0.97]^*^^†^	−0.79 [−1.00 to −0.58]			
S (W)	0–5	−19 [−36 to −1]	−63 [−80 to −45]^*^^†^	−32 [−51 to −13]	***p < 0.0001***	*p = 0.452*	*p = 0.569*
	5–10	−24 [−41 to −6]	−44 [−61 to −27]	−20 [−39 to −0.4]			
	10–15	−30 [−48 to −12]	−49 [−66 to −32]	−29 [−48 to −9]			
	0–15	−72 [−100 to −44]	−156 [−184 to −129]^*^^†^^*b*^	−82 [−112 to −51]	***p < 0.0001***	***p = 0.005***	*p = 0.097*
	0–60	−58 [−86 to −31]	−87 [−115 to −60]	−62 [−93 to −32]			
HR (bts·min^−1^)	0–5	−47 [−52 to −42]	−56 [−61 to −51]	−54 [−59 to −49]	*p = 0.339*	***p < 0.0001***	*p = 0.213*
	5–10	−7 [−12 to −2]^a^	−4 [−9 to 1]^a^	−4 [−9 to 2]^a^			
	10–15	−3 [−8 to 2]^a^	−6 [−11 to −1]^a^	−6 [−12 to −1]^a^			
	0–15	−57 [−65 to −50]^b^	−67 [−74 to −60]^*^^b^	−64 [−72 to −57]^*^^b^	***p < 0.0001***	***p < 0.0001***	*p = 0.473*
	0–60	−69 [−76 to −62]	−76 [−83 to −69]^*^	−72 [−79 to −65]			
TS	0–5	−0.9 [−1.1 to −0.6]	−1.0 [−1.3 to −0.8]	−1.0 [−1.2 to −0.8]	*p = 0.426*	***p < 0.0001***	*p = 0.993*
	5–10	−0.3 [−0.6 to −0.1]^a^	−0.5 [−0.7 to −0.2]^a^	−0.5 [−0.7 to −0.2]^a^			
	0–15	−0.2 [−0.5 to −0.03]^a^	−0.3 [−0.5 to −0.01]^a^	−0.3 [−0.5 to −0.05]^a^			
	10–15	−1.4 [−1.8 to −1.0]^b^	−1.8 [−2.2 to −1.4]^b^	−1.8 [−2.2 to −1.4]^b^	*p = 0.186*	***p < 0.0001***	*p = 0.906*
	0–60	−2.3 [−2.7 to −1.9]	−2.6 [−3.0 to −2.2]	−2.5 [−2.9 to −2.1]			

*Mixed Linear Model Covariates: T_re_: 38.29°C; ${\bar{T}}_{sk}$: 37.32°C; ${\bar{T}}_{b}$: 38.09°C; HR: 159 (bts·min^−1^); TS: 6.8; S: 87 W*.

a *Significantly (p < 0.05) different than 0–5 min*.

b *Significantly (p < 0.05) different than 0–60 min*.

* *Significantly different than control (p < 0.05)*.

† *Significantly different than single-hand cooling (p < 0.05)*.

*Bold values represent significant (p < 0.05) main and/or interaction effects*.

**Figure 2:**
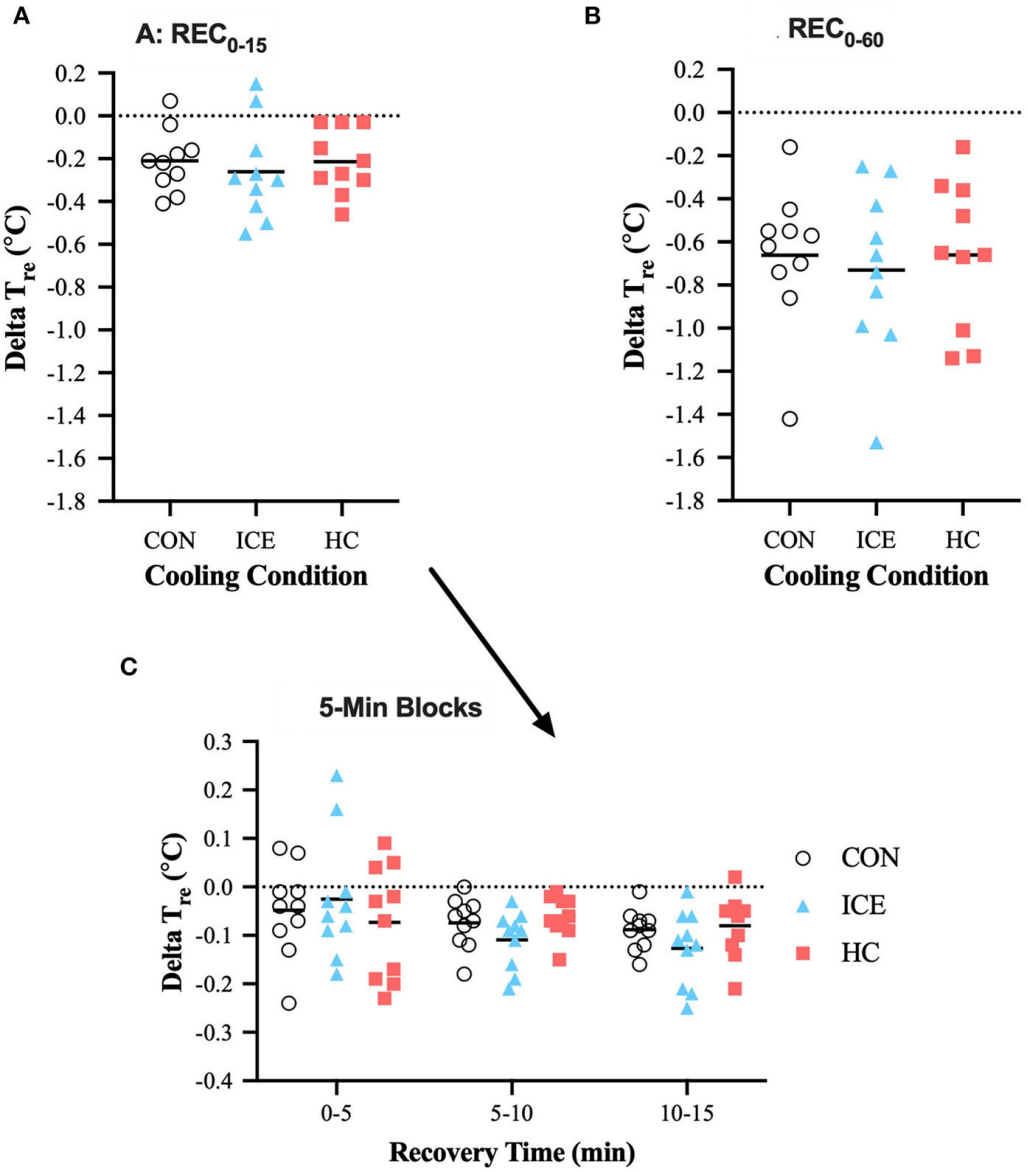
Mean (bar) and individual delta rectal temperature (*T*_re_) during REC_0−15_ **(A)**, REC_0−60_ **(B)**, and 5-min blocks during REC_0−15_ **(C)** when using ice vest (ICE), single-hand cooling (HC), and non-cooled control (CON) during heated exercise recovery.

No differences between conditions existed with regards to *T*_re_ reduction across time-limited or prolonged recovery (*p* = 0.990). Across all conditions, *T*_re_ recovery was greater during REC_0−60_ compared to REC_0−15_ (control: −0.45 [−0.67 to −0.24]°C, *p* < 0.0001; ice vest: −0.47 [−0.69 to −0.25]°C, *p* < 0.0001; single-hand cooling: −0.45 [−0.67 to −0.23]°C, *p* < 0.0001), which was expected given the difference in total recovery length. However, *T*_re_ recovery during REC_0−15_ accounted for 36% of the total *T*_re_ reduction during the full 60 min recovery, while it only accounted for 32% of *T*_re_ recovery for the control and single-hand cooling conditions. Interestingly, *T*_re_ recovery with the ice vest condition was significantly larger across both 5–10 min (−0.09 [−0.17 to −0.002]°C, *p* = 0.042) and 10–15 min (−0.10 [−0.18 to −0.02]°C, *p* = 0.009) of recovery compared to the first 0–5 min of recovery, indicating an initial delay in the ability of an ice vest to reduce *T*_re_. Mean delta and individual data for *T*_re_ is visualized in Table 1 and Figure 2, respectively.

**Figure 3:**
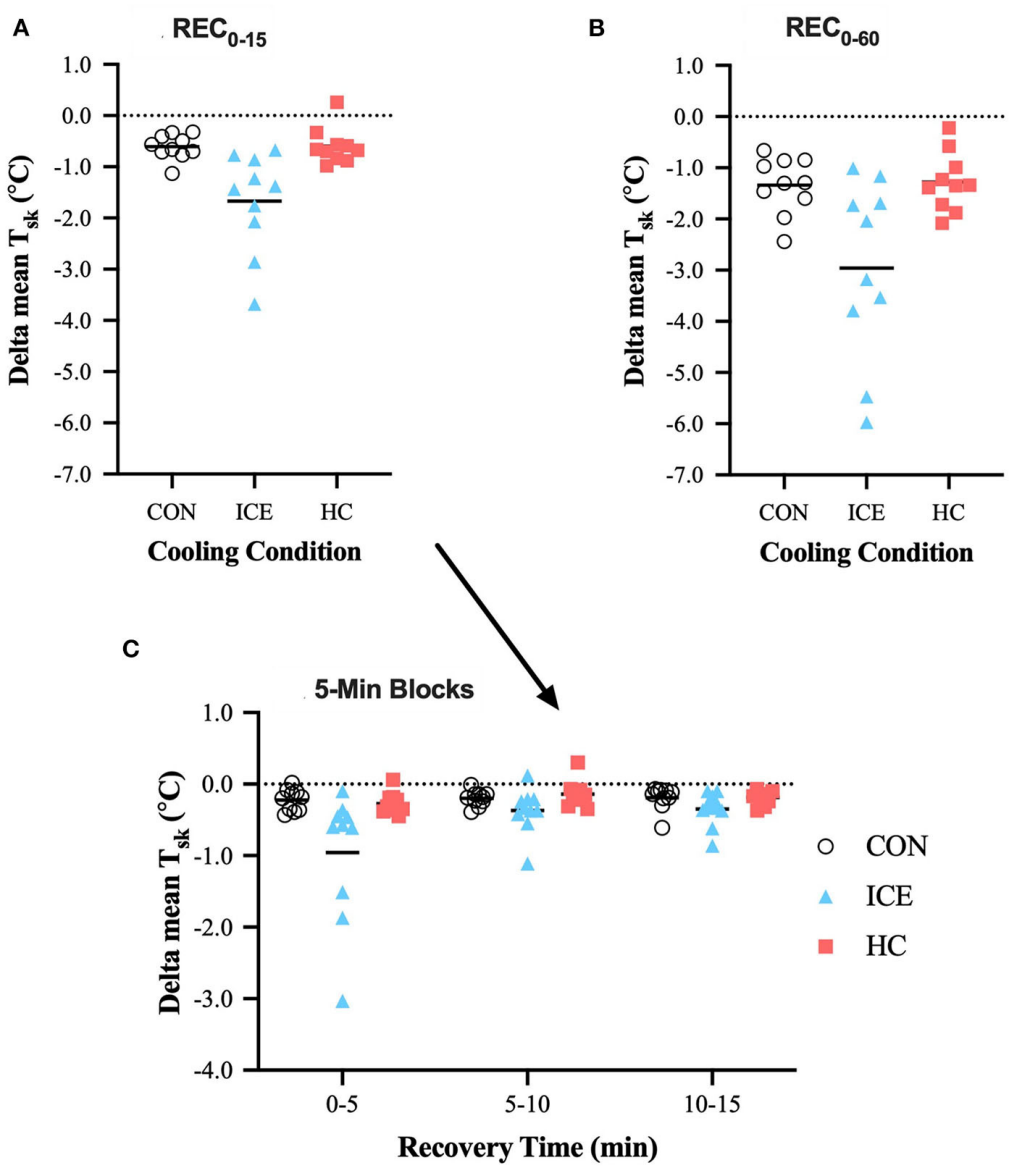
Mean (bar) and individual delta mean skin temperature (T_sk_) during REC_0−15_ **(A)**, REC_0−60_ **(B)**, and 5-min blocks during REC_0−15_ **(C)** when using ice vest (ICE), single-hand cooling (HC), and non-cooled control (CON) during heated exercise recovery.

During time-limited recovery, the ice vest was more effective at reducing ${\bar{T}}_{\text{sk}}$ compared to both control (−1.07 [−2.00 to −0.13]°C, *p* = 0.021) and single-hand cooling (−1.07 [−2.01 to −0.14]°C, *p* = 0.020). More specifically, the ice vest heightened recovery of ${\bar{T}}_{\text{sk}}$ within the first 5 min of recovery compared to both control (−0.74 [−1.12 to −0.36]°C, *p* < 0.0001) and single-hand cooling (−0.69 [−1.07 to −0.30]°C, *p* < 0.0001). The first 5 min of recovery appear to be especially important for the ${\bar{T}}_{\text{sk}}$ reducing effects of the ice vest, as ${\bar{T}}_{\text{sk}}$ was reduced more in the first 5 min as compared to 5–10 (−0.59 [−0.97 to −0.21]°C, *p* = 0.001) or 10–15 (−0.61 [−0.99 to −0.23]°C, *p* = 0.001) minutes. ${\bar{T}}_{\text{sk}}$ was also more effectively reduced with ice vest application when considering prolonged exercise recovery (REC_0−60_) compared to both control (−1.62 [−2.56 to −0.68]°C, *p* < 0.0001) and single-hand cooling (−1.68 [−2.61 to −0.74]°C, *p* < 0.0001). Although, the ice vest was efficient in reducing ${\bar{T}}_{\text{sk}}$ within the first 15 min of recovery, considerable further skin cooling was apparent with extending the length of vest cooling exposure (REC_0−60_). For the ice vest condition, prolonged recovery elicited greater reductions in ${\bar{T}}_{\text{sk}}$ compared to time-limited recovery (−1.29 [−2.05 to −0.53]°C, *p* = 0.001). However, ${\bar{T}}_{\text{sk}}$ recovery during REC_0−15_ accounted for 56% of the total ${\bar{T}}_{\text{sk}}$ recovery during the full 60 min recovery for the ice vest, while it only accounted for 46% of ${\bar{T}}_{\text{sk}}$ recovery for control and 47% for single-hand cooling. Mean delta and individual data for ${\bar{T}}_{\text{sk}}$ can be found in Table 1 and Figure 3, respectively.

**Figure 4:**
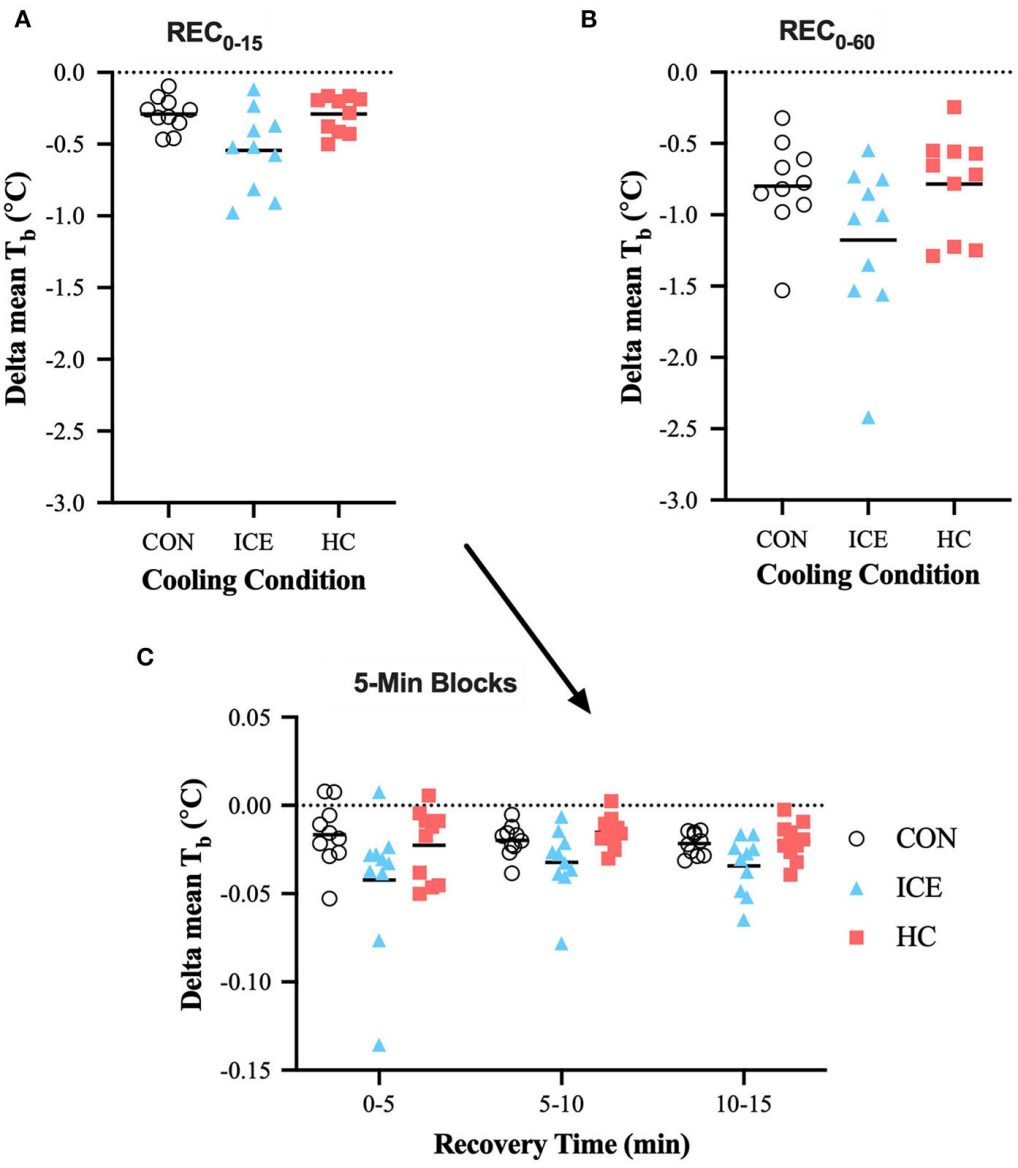
Mean (bar) and individual delta mean body temperature (T_b_) during REC_0−15_ **(A)**, REC_0−60_ **(B)**, and 5-min blocks during REC_0−15_ **(C)** when using ice vest (ICE), single-hand cooling (HC), and non-cooled control (CON) during heated exercise recovery.

Considering the entire recovery period (REC_0−60_), an ice vest was able to more effectively recover ${\bar{T}}_{\text{b}}$ compared to both control (−0.38 [−0.69 to −0.07]°C, *p* = 0.012) and single-hand cooling (−0.39 [−0.70 to −0.08]°C, *p* = 0.010), although this could not confidently be extended to the time-limited recovery window (REC_0−15_). Considering the components of the time-limited recovery, an ice vest was also able to more effectively reduce ${\bar{T}}_{\text{b}}$ compared to control specifically during the first 5 min of recovery (−0.13 [−0.23 to −0.03]°C, *p* = 0.008), likely the result of ${\bar{T}}_{\text{sk}}$ reductions. Across all conditions, ${\bar{T}}_{\text{b}}$ was recovered to a greater extent with prolonged recovery compared to time-limited recovery (control: −0.51 [−0.76 to −0.26]°C, *p* < 0.0001; ice vest: −0.63 [−0.89 to −0.38]°C, *p* < 0.0001; single-hand cooling: −0.50 [−0.75 to −0.24]°C, *p* < 0.0001). However, ${\bar{T}}_{\text{b}}$ recovery during REC_0−15_ accounted for 46% of the total ${\bar{T}}_{\text{b}}$ recovery during REC_0−60_ for the ice vest, while it only accounted for 36% of ${\bar{T}}_{\text{b}}$ recovery for control and 37% for single-hand cooling. Mean delta and individual data for ${\bar{T}}_{\text{b}}$ can be found in Table 1 and Figure 4, respectively.

Single-hand cooling did not display any significant differences compared to a non-cooling control or ice vest with regards to change in S, ${\bar{T}}_{\text{b}}$, *T*_re_, or ${\bar{T}}_{\text{sk}}$ at REC_0−15_ or REC_0−60_ of post exercise recovery.

#### Heart Rate

**Figure 5:**
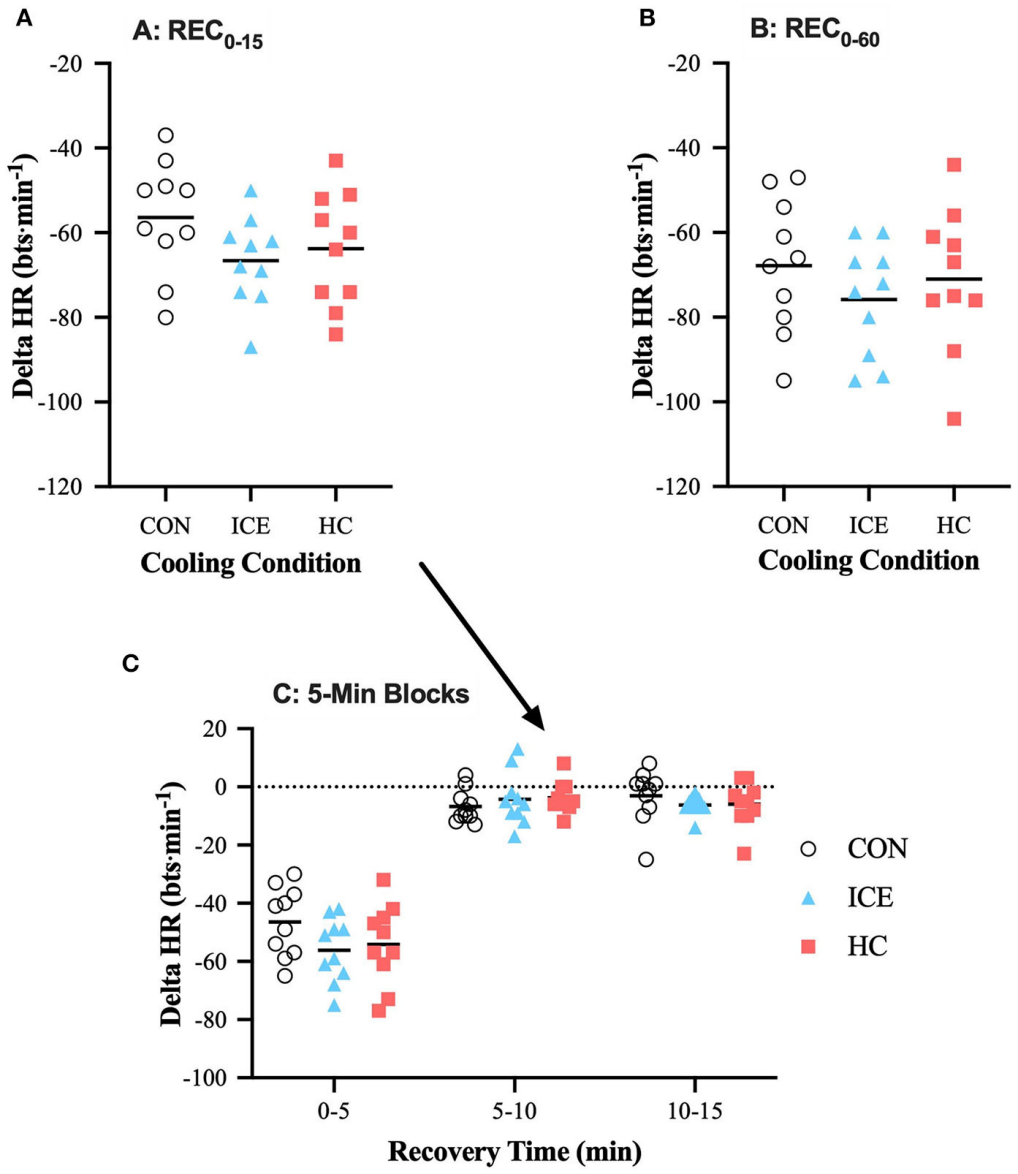
Mean (bar) and individual delta heart rate (HR) during REC_0−15_ **(A)**, REC_0−60_ **(B)**, and 5-min blocks during REC_0−15_ **(C)** when using ice vest (ICE), single-hand cooling (HC), and non-cooled control (CON) during heated exercise recovery.

Across all conditions, HR recovery was greatest during the first 5 min of recovery compared to the subsequent 10 min but was overall greater during REC_0−60_ vs. REC_0−15_ as a function of time (see Tables 1, 2). During time-limited recovery (REC_0−15_) both the ice vest (−9 [−15 to −3] bts·min^−1^, *p* = 0.002) and single-hand cooling (−7 [−13 to −1] bts·min^−1^, *p* = 0.021) were able to recover HR more effectively compared to control however, only the ice vest was able to recover HR more effectively compared to control (−7 [−13 to −1] bts·min^−1^, *p* = 0.029) during the prolonged recovery window (REC_0−60_). Mean delta and individual data for HR is visualized in Table 1 and Figure 5, respectively.

**Table 2:**
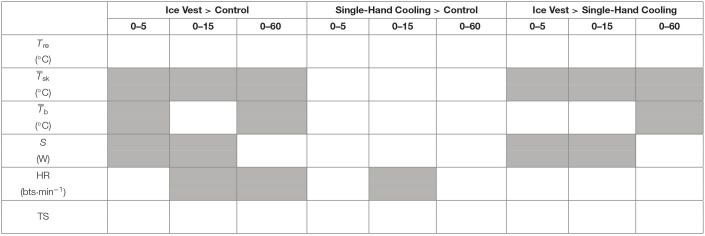
Visual summary of thermo-physiological and thermal perceptual differences between cooling conditions across immediate (REC_0−5_), time-limited (REC_0−15_), and prolonged recovery (REC_0−60_).

*Gray boxes indicate significant difference (p <0.05) for the column described difference in recovery magnitude; White boxes indicate a lack of significant difference (p > 0.05) for the column described difference in recovery magnitude; 0–5, 0–5 min of recovery; 0–15, 0–15 min of recovery; 0–60, 0–60 min of recovery*.

#### Perceptual Index

Across all conditions, reductions in TS were greatest during the first 5 min of recovery compared to the subsequent 10 min but were overall greater during REC_0−60_ vs. REC_0−15_ as a function of time. No differences between conditions existed with regards to TS reduction across time-limited or prolonged recovery (*p* = 0.906). Mean delta data for TS is presented in Table 1.

#### Body Fluid Balance

Pre-post fluid loss, as indicated by change in nude body mass, was similar across the three trials (single-hand cooling: 1.19 ± 0.56%, ice vest: 1.27 ± 0.47%, non-cooling control: 1.40 ± 0.68%; *p* = 0.490).

## Discussion

The primary aim of this study was to investigate the effectiveness of time-limited and long-term active cooling compared to passive recovery following prolonged submaximal exercise in the heat with sport-mimicking recovery conditions. Neither the ice vest nor single-hand cooling were able to improve *T*_re_ or TS recovery at any time post-exercise compared to a non-cooling control. However, during time-limited recovery an ice vest induced greater recovery of ${\bar{T}}_{\text{sk}}$ and improved heat loss compared to a non-cooling control, both of which were apparent within the first 5 min of recovery. Reductions in ${\bar{T}}_{\text{sk}}$ were also greater with an ice vest compared to a non-cooling control when considering the prolonged 60 min recovery (REC_0−60_), translating into greater reductions in calculated mean body temperature. Single-hand cooling was unable to augment recovery of ${\bar{T}}_{\text{b}}$, S, or ${\bar{T}}_{\text{sk}}$ compared to a non-cooling control with any investigated recovery length. Yet, both the ice vest and single-hand cooling enhanced HR recovery compared to a non-cooling control early during exercise recovery (REC_0−15_), although this remained distinguishable into prolonged recovery (REC_0−60_) only for the ice vest (see Table 2).

A secondary aim of this study was to examine thermo-physiological and perceptual differences during heated recovery between negative pressure hand cooling and the application of an ice vest. An ice vest was no more effective at reducing *T*_re_, TS, or HR compared to single-hand cooling. That said, compared to single-hand cooling, an ice vest more effectively reduced ${\bar{T}}_{\text{sk}}$ during both time-limited (REC_0−15_) and prolonged recovery (REC_0−60_). The time-limited recovery reduction in ${\bar{T}}_{\text{sk}}$ elicited by an ice vest concurrently enhanced heat loss compared to single-hand cooling (see Table 2).

### Exercise Recovery: Ice Vest Influence on Thermoregulatory Recovery

Wearing an ice vest during time-limited heated exercise recovery enhanced heat loss and ${\bar{T}}_{\text{sk}}$ reductions compared to a non-cooling control The application of 0°C ice packs to the temperature elevated skin provided a widened heat loss gradient that facilitated greater heat loss from the blood perfusing the skin. Due to the 30% contribution of the chest thermocouple to the ${\bar{T}}_{\text{sk}}$ calculation (Ramanathan, 1964), the enhanced reduction of ${\bar{T}}_{\text{sk}}$ may be a more accurate representation of the change in chest skin temperature than a change in whole body skin temperature. Despite this influence, a lowered *T*_sk_ at the chest likely still has an impact on thermoregulatory function via peripheral thermoregulatory mechanisms (Huizenga et al., 2004).

Interestingly, the improvements in ${\bar{T}}_{\text{sk}}$ reduction by the phase-changing ice vest were not able to translate to a significantly greater recovery of *T*_re_ compared to a non-cooling control during heated exercise recovery. Similar investigations support this notion that phase-changing cool inserts may be effective at cooling the skin but tend to have a substantially smaller effect on measured deep body temperature (Duffield and Marino, 2007; Barwood et al., 2009). One possible explanation lies in the large surface area covered by the vest (~26% of total body surface area) which may impede immediate post-exercise evaporative cooling that would otherwise contribute to reductions in deeper body temperature (Barwood et al., 2009). As subjects were cooled by the ice vest, so too the ice vest was warmed by the subject and ambient environment, further reducing heat loss gradients and therefore cooling magnitude over time. Cooling investigations using temperature-maintained liquid perfused vests suggest they may be more viable to manipulate deeper body temperature (Balldin et al., 2007; Amorim et al., 2010), however it may be argued that maintenance of water temperature or other liquid-perfused vests may be logistically difficult, especially when attempting to cool in thermally-challenging environmental conditions. Despite the core temperature independent perceptual link between TS and localized ${\bar{T}}_{\text{sk}}$ (Schlader et al., 2011), TS was also not significantly altered by the ice vest. Personal subjective bias resulting from differences in heat tolerance and psychological bias may lead to overestimation of thermal relief upon cessation of exercise.

Wearing an ice vest reduced HR beyond that of a non-cooling control during both time-limited and prolonged heated recovery. A reduction in peripheral vasodilation, due to ice vest application, likely results in an increase in central venous pressure via a shift of cutaneous blood into the thoracic vasculature. This shift simultaneously stimulates high arterial pressure and low cardiopulmonary pressure baroreflexes, effectively eliciting an increase in cardiac vagal tone (Pump et al., 2001) and resultant expedited decrease in HR beyond that demonstrated by passive exercise recovery. This hypothesis along with the much smaller *T*_re_ recovery within the first 5 min of recovery for the ice vest further supports the notion that reactive peripheral vasoconstriction, depending on the magnitude as reflective of cold severity, may to some extent reduce the immediate cooling capability of the ice vest. However, it may be argued that the phase-changing nature of the ice vest reduces the impact of this initial vasoconstrictive clamping as the ice packs melt. Similarly to the ice vest, single-hand cooling was able to reduce HR beyond that of the non-cooling control after 15 min of exposure. This is to some extent surprising, but may indicate that even single hand cooling is sufficient to initiate reflexive baroreceptor controlled increases in cardiac vagal tone.

An ice vest was superior to single-hand cooling in reducing ${\bar{T}}_{\text{sk}}$ during REC_0−5_, REC_0−15_, and REC_0−60_ and heat storage during time-limited recovery. This may be explained by the ice vest's greater surface area (~26 vs. 1% of total body surface area), upon which the widened cooling gradient is applied. Further evaluation of the rate of *T*_re_ recovery for all conditions, especially during the first 5 min of recovery, indicates that single-hand cooling may be the quickest, of the strategies tested, to initially reduce *T*_re_ (single-hand cooling: −0.015°C·min^−1^, ice vest: −0.007°C·min^−1^, non-cooling control: −0.010°C·min^−1^). The rate of cooling by single-hand cooling in the present study is similar to that reported in previous literature investigating cooling of one hand (−0.017 °C·min^−1^) (Grahn et al., 2009). The comparatively small rate of *T*_re_ change with the ice vest supports the likelihood of reactive cutaneous vasoconstriction and/or a sudden reduction in evaporative cooling due to the surface area covered by vest application. Two subjects (Figure 2) demonstrated continued rise in *T*_re_ despite 5 min of vest application indicating that although a quick change in skin temperature did tend to occur within the first 5 min (Figure 3) it likely contributed to some degree of vasoconstriction and/or lack of evaporative cooling capability. While an ice vest was slower to recover *T*_re_ immediately upon application compared to single-hand cooling and a non-cooling control, the ice vest demonstrated superior cooling rates at both 5–10 (single-hand cooling: −0.012°C·min^−1^, ice vest: −0.022°C·min^−1^, non-cooling control: −0.015°C·min^−1^) and 10–15 min (single-hand cooling: −0.017°C·min^−1^, ice vest: −0.025°C·min^−1^, non-cooling control: −0.018°C·min^−1^). Combined with the larger influence of an ice vest on thermo-physiological responses during heated exercise recovery, these rates indicate that, of the methods tested, an ice vest may be the most applicable to achieve effective cooling during time-limited recovery.

### Exercise Recovery: Single-Hand Cooling Influence on Thermoregulatory Recovery

Single-hand cooling using a negative pressure device was unable to enhance heated exercise thermoregulatory recovery beyond that of the non-cooling control regardless of application length (15 vs. 60 min). A similar investigation capable of minimally increasing *T*_re_ to 37.72°C also indicated a single-hand cooling device was no more effective at decreasing *T*_re_ following heated (35°C, 85% RH) exercise than control or vest conditions (Balldin et al., 2007). This minor thermal gain likely stimulated a smaller volume of blood to the skin providing a very small heat dissipation gradient, thereby minimizing the influence of the hand-cooling device. Still, even with much greater thermoregulatory stress, the result of both a higher ambient temperature (42°C) and heat loss retardant clothing, negative pressure single hand cooling was no more effective at deep body temperature recovery following exercise (*T*_re_ 38.5°C) than a non-cooling control (Amorim et al., 2010). The significantly lower body surface area cooled with single-hand cooling vs. a torso-encompassing vest may help to explain single-hand cooling's thermoregulatory shortcomings as both the present study and Amorim et al. indicate vest application, a phase-changing ice and cold water perfused vest, respectively, as a superior cooling technique. The unique presence of a continued environmental heat stress during recovery with the cooling device may therefore reduce the overall likelihood of single-hand cooling to enhance core temperature recovery.

Core temperature at cessation of exercise, and therefore start of exercise recovery, likely also plays a key role in the efficacy of single-hand cooling as a thermoregulation enhancing device. As both core and skin temperatures tend to rise commensurately (Gleeson, 1998), a greater achieved core temperature is likely reflective of a greater skin temperature, effectively providing a widened heat loss gradient when a cooling device is applied that favors improved heat loss. Firefighters recovering from heavy physical work in 36°C, 44% RH performed to a *T*_re_ of 39°C cooled 144% more with single-hand cooling for 40 min compared to passive cooling (Zhang et al., 2009). Furthermore, individuals heated using 39.3°C 38% RH ambient conditions paired with vigorous walking to a *T*_re_ of 39.44°C experienced enhanced *T*_re_ recovery following a 20 min application of an ~17°C cooling device on each hand (Adams et al., 2016). Barwood et al. (2009) additionally investigated a two-hand cold water immersion exercise recovery strategy that induced heat loss of ~162 W after 10–15 min and was effective at augmenting reduction in *T*_re_ compared to a non-cooling control condition (heat loss ~99 W). Heat loss induced via this dual hand cold water immersion is similar to that induced by the ice vest in the current investigation over a similar 15 min time period, ~166 W, and yet the ice vest was unable to elicit enhanced *T*_re_ recovery. As heat storage is determined utilizing a change in ${\bar{T}}_{\text{b}}$, it appears that dual hand cooling and ice vest application may modulate calculated body temperature divergently within the first 15 min of cooling, with dual hand cooling having greater influence on *T*_re_ hypothesized to result from significant cooling of the blood, without significant change in ${\bar{T}}_{\text{sk}}$ and vice versa for ice vest application.

Single-hand cooling in our investigation induced heat loss of ~84 W, roughly half that demonstrated by dual-handed immersion at the same ~17°C exposure temperature (Barwood et al., 2009), over the first 15 min of recovery, indicating the importance of maximizing the amount of surface area cooled to thermoregulatory recovery. Additionally as hand water immersion sans the use of a negative pressure vacuum seal was successful in augmenting *T*_re_ recovery, the necessity or usefulness of this feature to offset otherwise inhibiting vasoconstriction is thought questionable. Overall, literature suggests the influence of cooling with or without a vacuum on heat loss appears small (~12 W) (Kuennen et al., 2010). Ultimately, the cooling effectiveness of cold water hand immersion is determined by the maintenance of peripheral blood flow as well as the heat loss gradient magnitude between the skin and the immersion water. Water of ~15°C is sufficient to induce peripheral vasoconstriction in individuals with maintained deep body temperatures (Tipton et al., 1993), rendering the selected hand cooling temperature of the current study near optimal. Collectively, literature that has been successful at modulating *T*_re_ during recovery in the heat beyond that possible by passive cooling demonstrates that hand cooling may be capable of greater cooling compared to a non-cooling control. It does appear that a few conditions may be necessary to facilitate: (1) *T*_re_ of ~39°C that facilitates a larger heat loss gradient and reduces the influence of peripheral vasoconstriction; and (2) the use of dual hand vs. single hand cooling to maximize the amount of total body surface area cooled.

Methodological limitations to measurement of thermoregulatory variables must also be considered. Post-exercise *T*_re_ values indicate a similar level of thermal stress between our three separate trial conditions. Similar literature places this exercise thermal stress in a “moderately high” category. Other investigations, utilizing *T*_re_, have successfully produced a larger thermal gain, achieving temperatures as high as 39–39.44°C (Zhang et al., 2009; Adams et al., 2016). These temperatures were set as exercise end-points rather than a product of a given duration of exercise time and were accomplished with a combination of both environmental and clothing manipulation. We chose instead to impose a time relative end-point to exercise (60 min) for two reasons: (1) The subject population recruited consisted primarily of natives to a variable climate region with little exposure to the experience of performing exercise in considerable heat. For this reason, two preliminary subjects exhibited significant difficulty in completing the exercise task to a core temperature >38.5°C; (2) In a real-world scenario, the likelihood of athletes or occupational workers performing the same duration of physical work is much greater than achieving an identical exercise core temperature. Numerous investigators have provided data to suggest the inability of *T*_re_ to respond as readily to rapid changes in core temperature compared to esophageal temperature (Lee et al., 2000; Easton et al., 2007). While subjects wore heavy heat retardant uniforms after exercising in the heat to an esophageal temperature of 38.8°C, a single-hand cooling device elicited significantly lower esophageal and skin temperatures from 15 to 50 min of exercise recovery (Kuennen et al., 2010). Esophageal temperatures may offer improved sensitivity and responsiveness over rectal temperatures, especially as rate of cooling is prioritized. Esophageal temperature, though, does pose significant practicality concerns as many subjects struggle to place and tolerate the temperature probe. Due to logistical difficulties with efficiently replicating wind velocity, it was omitted from the current design. Wind velocity should, however, be considered as a variable that may reduce or otherwise alter the efficiency of the investigated cooling devices.

## Conclusions

Wearing an ice vest but not single hand-cooling, using a cold water low pressure vacuum device, was effective at reducing thermo-physiological strain during both time-limited and prolonged heated exercise recovery. When a prolonged heated recovery time is accessible, an ice vest is superior to both passive and single-hand cooling to reduce mean body and skin temperatures. An ice vest also appears efficacious in reducing mean skin temperature after as little as 15 min of application, making its use specifically advantageous for time-limited sport or occupational recovery. Although the lower mean skin temperatures after 15 and 60 min were not able to elicit a significantly lower *T*_re_ or a reduced thermal strain, this peripheral activity may still impact overall thermoregulatory function. With moderately, rather than severely, elevated core temperatures, time-limited sport thermoregulatory recovery in the heat is likely enhanced with the use of techniques that encompass a larger degree of body surface area, like an ice vest. This may be particularly meaningful during sporting half-time to proactively offset progressive increases in thermal stress during a second-half physical effort. Further research is necessary to pinpoint the exact environmental conditions and level of hyperthermia for which single-hand cooling or phase-changing ice vest application may be most beneficial.

## Data Availability Statement

The raw data supporting the conclusions of this article will be made available by the authors, without undue reservation.

## Ethics Statement

The studies involving human participants were reviewed and approved by Human Subjects Institutional Review Board Western Michigan University. The patients/participants provided their written informed consent to participate in this study.

## Author Contributions

AS performed the experiments and collected all participant data. AS and RS conceived and designed the experiments, analyzed the data, summarized the results, wrote and revised the manuscript. All authors contributed to the article and approved the submitted version.

## Conflict of Interest

The authors declare that the research was conducted in the absence of any commercial or financial relationships that could be construed as a potential conflict of interest.
